# 1-L Transcription in Prion Diseases

**DOI:** 10.3390/ijms25189961

**Published:** 2024-09-15

**Authors:** Jozef Nahalka

**Affiliations:** 1 Centre for Glycomics, Institute of Chemistry, Slovak Academy of Sciences, Dubravska Cesta 9, SK-84538 Bratislava, Slovakia; nahalka@savba.sk; 2 Centre of Excellence for White-Green Biotechnology, Slovak Academy of Sciences, Trieda Andreja Hlinku 2, SK-94976 Nitra, Slovakia

**Keywords:** prion diseases, post-transcriptional regulation, protein-RNA recognition, bioinformatics method, identified genes

## Abstract

Understanding the pathogenesis and mechanisms of prion diseases can significantly expand our knowledge in the field of neurodegenerative diseases. Prion biology is increasingly recognized as being relevant to the pathophysiology of Alzheimer’s disease and Parkinson’s disease, both of which affect millions of people each year. This bioinformatics study used a theoretical protein-RNA recognition code (1-L transcription) to reveal the post-transcriptional regulation of the prion protein (PrP^C^). The principle for this method is directly elucidated on PrP^C^, in which an octa-repeat can be 1-L transcribed into a GGA triplet repeat RNA aptamer known to reduce the misfolding of normal PrP^C^ into abnormal PrP^Sc^. The identified genes/proteins are associated with mitochondria, cancer, COVID-19 and ER-stress, and approximately half are directly or indirectly associated with prion diseases. For example, the octa-repeat supports CD44, and regions of the brain with astrocytic prion accumulation also display high levels of CD44.

## 1. Introduction

1-L transcription is a recently introduced and very simple bioinformatics method [1,2,3,4,5] that uses a theoretical protein–RNA recognition code [6,7]. This method is based on the principle that RNA binding proteins (RBPs) use at least one amino acid sequence that can be 1-L transcribed into the exact nucleotide sequence of the recognized RNA. 1-L transcription can thus be used to identify genes/proteins that are post-transcriptionally regulated by the RBP. To illustrate the 1L transcription method, the alignment of 1-L transcribed RBP GEMIN5 (gem nuclear organelle associated protein 5) and RNU2-1 (U2 spliceosomal RNA) was presented recently as an example of such an RBP [1].

**Figure 1:**
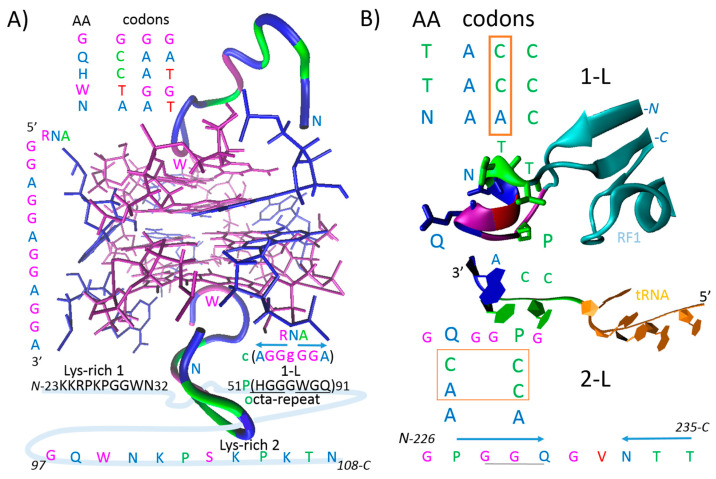
A theoretical protein–RNA recognition code. (**A**) GGA triplet repeat RNA (R12) in complex with Lys-rich 2 peptide of the prion protein. RNA aptamer forms a dimer and each monomer binds one Lys-rich 2 peptide. The tryptophan residues (TGG codon) are oriented to the guanosine core (magenta color) and asparagine residues (AAT codon) to adenines (blue color). The 1-L transcription of the octa-repeats is compatible with the GGA triplet repeat RNA. (**B**) The ribosomal release factor 1 (RF1) interacting with P-site tRNA. 1-L, one-letter code—second nucleotide in codons; 2-L, two-letter code—first two nucleotides in codons [6]. At a shorter distance, proline 227 (P) and glutamine 230 (Q) recognize terminal CCA3′ nucleotide sequence using the 2-L code; at a longer distance, asparagine 233 (N), threonine 234 (T) and T235 recognize terminal CCA3′ nucleotide sequence using 1-L code in the reversed mode. Interestingly, these two sequences are spaced with glycine (G) and valine (V) which can be 2-L transcribed to the complementary sequence GGU5′. The coordinates were downloaded from Protein Data Bank; 2RSK (**A**) and 4V63 (**B**) are the corresponding PDB codes, and Visual Molecular Dynamics (VMD 1.9.3) was used for the visualization.

Liquid–liquid phase separation (LLPS) has emerged as a mechanism for the dynamic and reversible assembly of ribonucleoprotein complexes and is a major factor in the mesoscale organization of proteins and RNAs. Consequently, the misregulation of biomolecular condensates leads to the formation of insoluble aggregates in several neurodegenerative diseases [8]. The sequence-specific RBP–RNA interactions are generally maintained inside the condensates [9]. RNA molecules are increasingly recognized as important factors contributing to ‘prion behavior’ [10]. For example, the prion protein (PrP^C^) interacts with and acts as a “chaperone” for HIV-1 genomic RNA [11]. More recently, PrP^C^ was shown to have antiviral activity against infection with Japanese encephalitis virus and may also be a promising therapeutic target for flavivirus infections (e.g., Zika virus, dengue virus, yellow fever virus) [12]. Interestingly, it was reported 15 years ago that an RNA aptamer (and to a much lesser extent DNA aptamer) possessing four GGA triplet repeats (R12) could reduce the PrP^C^ aggregation level in mouse neuronal cells that were persistently infected with a transmissible spongiform encephalopathy agent [13]. Two lysine-rich sequences in PrP^C^ (Lys-rich 1/2) were identified as binding sites for R12 and provide the electrostatic interaction between the uniquely arranged phosphate groups of R12 and the lysine clusters of peptides [14]. The interaction between Lys-rich 2 and R12 was studied and deposited in the protein data bank (2RSK, Figure 1A) [13]. The LLPS of PrP^C^ is regulated by the octa-repeat domain independently of histidine residues and copper [15], while sequence-specific RBP–RNA interactions are generally maintained inside condensates [9]. Hence, the octa-repeat domain is important not only for the LLPS, but also for the specific protein-RNA recognition of PrP^C^ (Figure 1A).

During the evolution of the genetic code in the early prebiotic stage, there was likely a stereo-chemical era involving direct interactions between RNA nucleotides and peptide amino acids. In theory, this has been performed in LLPS condensates. Following the crystal structure of aminoacyl transfer RNA synthetases that interact with tRNA, it was proposed the protein–RNA recognition code could be derived from the present genetic code [6]. The principle of the protein–RNA recognition code is explained in Figure 1B and is depicted in the crystal structure of ribosomal release factor 1 (RF1), which interacts with P-site tRNA (4V63). In the type I release factors of all organisms, glutamine 230 in the GGQ motif (a universally conserved motif) contributes directly to peptidyl-tRNA hydrolysis and translation termination on the 70S ribosome [16]. Q_230_, which has a CAA codon, recognizes and interacts with the C_2452_A_2451_ of rRNA and the terminal adenine of tRNA [16]. In the RF1 of *Thermus thermophilus*, in addition to Q_230_, the proline 227 (P_227_) and NTT (asparagine–threonine–threonine) motif help to recognize the CCA-end of tRNA (Figure 1B). The CCA_76_ readout is performed using the CC and CA readout-2-letter code (2-L), or using the C plus C plus A readout-1-letter code (1-L), as proposed earlier [6,7]. As shown in Figure 1B, the readout of the RNA by the peptide can occur in both directions: N to C, and C to N. In PrP^C^, an octa-repeat composed of glycine residues overlapped by histidine and glutamine residues can be 1-L transcribed into the GGA RNA repeat motif (Figure 1A). Unsurprisingly, the R12 GGA aptamer was identified as a ligand that reduces the level of PrP^C^ aggregation in mouse neuronal cells infected with transmissible spongiform encephalopathy [13]. Interestingly, the same GGQ amino acid motif was proposed for the recognition of ribose in prebiotic history, with ribose being the sugar component of RNA [17]. It is also interesting that lysine and asparagine in the Lys-rich sequences of PrP^C^ (Figure 1A) have AAA/G and AAT/C codons, respectively. Polyadenosine RNA has been proposed as a possible agent that increases PrP^C^ aggregation [18]. In summary, sequence-specific RBP–RNA interactions in neurons are maintained inside LLPS-condensates [9] and preserve the protein-RNA recognition code [6]. Moreover, more than 1300 proteins present in the lysate of human neurons were found to be maintained in a soluble and functional state by associating with endogenous RNA, since RNA degradation invariably leads to protein aggregation. The majority of these proteins lack conventional RNA-binding domains [19].

The prion disease-associated transmissible agent was named as a ‘prion’ (proteinaceous infectious) particle because it appeared to be devoid of any nucleic acid and consisted of aggregated protein only. Clinical symptoms of prion diseases include rapid cognitive decline and death, often within months [20]. The PrP^C^ protein is encoded by the PRNP gene. Prion diseases are associated with rare mutations of the PRNP gene (genetic forms) or rare transmission of the prion protein between humans and mammals (acquired forms). They can occur spontaneously (sporadically), with sporadic Creutzfeldt–Jakob disease (sCJD) being the most common form in humans. Although prion diseases are relatively rare, understanding their pathogenesis and mechanisms may significantly enhance knowledge in the field of neurodegenerative diseases. Indeed, prion biology is increasingly recognized as being relevant to the pathophysiology of Alzheimer’s disease and Parkinson’s disease, both of which affect millions of people every year [20].

**Figure 2:**
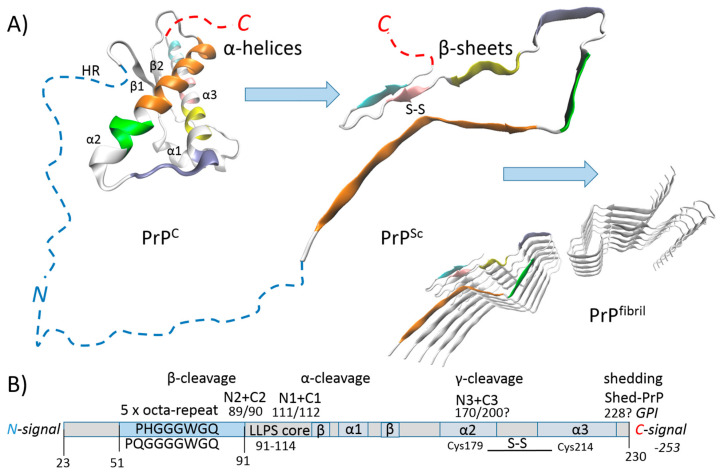
The prion protein. (**A**) Misfolding of the prion protein (PrP^c^) into PrP^Sc^. Misfolded PrP^Sc^ forms self-replicating protofibril (PrP^fibril^) core. PrP^fibril^ templates the native PrP^C^ form into PrP^Sc^ form and forms detergent/protease-resistant amyloid fibrils/aggregates/plaques in vivo. (**B**) Schematic domain representation of the prion protein and its α/β/γ-cleavage and shedding.

Prion diseases are caused by the misfolding of a normal α-helix-rich protein called cellular prion protein (PrP^C^) into an abnormal β-sheets-rich protein called PrP^Sc^, which then forms a self-replicating protofibril (PrP^fibril^) core (Figure 2A). PrP^fibril^ serves as a template for native PrP^C^ to form into PrP^Sc^, which is stabilized by a disulfide bond and collects into amyloid fibril-aggregate plaques in vivo that are detergent/protease-resistant [21,22]. Prion particles in the brain replicate in both astrocytes and neurons [23], whereas microglia are involved in the clearance of PrP^Sc^/PrP^fibril^ during early preclinical stages. During the late preclinical stage, the target of microglial activity shifts from PrP^Sc^ uptake to the engulfment of neurons. This transition in microglial phagocytic behavior is followed by the rapid accumulation of PrP^Sc^ [24].

PrP^C^ is a glycoprotein that exists in both membrane- and non-membrane-bound forms. It contains five glutamine/histidine-containing octa-peptide repeats (residues 51–91), an LLPS core (residues 91–114) and an α-helice-globular domain (residues 128–230) (Figure 2B). In healthy tissues, α/β/γ-cleavage of PrP^C^ results in signaling (Figure 2B). For example, the N1-fragment has a neuroprotective role by acting as a ligand for G protein-coupled receptor signaling in Schwann cells to support myelin maintenance, while also causing anti-apoptotic effects by activating p53 [25]. PrP^C^ is a GPI (glycosylphosphatidylinositol)-anchored protein that has an N-terminal signal peptide for ER localization and a C-terminal signal peptide for the attachment of GPI [26]. When PrP^C^ is GPI-anchored, the metalloprotease ADAM10 can release nearly full-length PrP^C^, referred to as “shed-PrP”, into the extracellular space [25]. However, the physiological roles of shed-PrP are yet to be fully studied. The LLPS of the mammalian prion protein is mainly driven by its LLPS-core. However, the ability to undergo LLPS has evolved with the octa-repeat in the intrinsically disordered domain, independently of the histidine residues [15]. Moreover, the histidine residues can be effectively substituted by glutamine residues (Figure 2B). In summary, the N1-fragment has a neuroprotective role inside biomolecular LLPS-condensates.

In summary, prion diseases are caused by the misfolding of a normal α-helix-rich PrP^C^ into an abnormal β-sheets-rich PrP^Sc^. PrP^C^ is an RBP and may have a neuroprotective role inside biomolecular condensates. Sequence-specific RBP–RNA interactions in neurons are maintained inside the LLPS-condensates and preserve the protein-RNA recognition code. This code can be used to identify genes/proteins that are post-transcriptionally regulated by the RBP. In the present study, PrP^C^ was 1-L transcribed into a nucleotide sequence and compared with the human transcriptome (BLASTn). The identified genes/proteins were comprehensively reviewed here, and their functions and profile were found to be consistent with prion diseases. However, this study is purely bioinformatic in nature and experimental validation is needed to confirm the in silico predictions.

## 2. Results and Discussion

**Figure 3:**
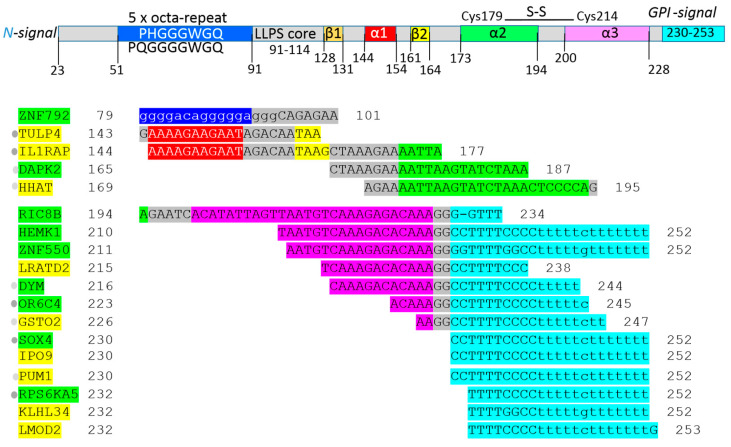
Identified genes/proteins via 1-L transcription of *N*-(AA)n-*C* sequence. The found sequences are colored according to the schematic domain architecture of the prion protein. α = alpha-helical domains (red, light green, magenta), β = beta-sheet stretches (yellow), 5 x octa-repeat sequence (blue) and GPI-signal sequence (cyan). A GPI (glycosylphosphatidylinositol) anchor is added at AA 230 as a post-translational modification. An LLPS core means that the main sequence participated in liquid–liquid phase separation. The green gene highlights show alignments with the gene transcript RNA sequence (post-transcriptionally repressed) and yellow gene highlights show alignments with the reverse complement RNA sequences (post-transcriptionally promoted). The identified genes/proteins can be directly (●) or indirectly (●) linked to prion diseases.

**Figure 4:**
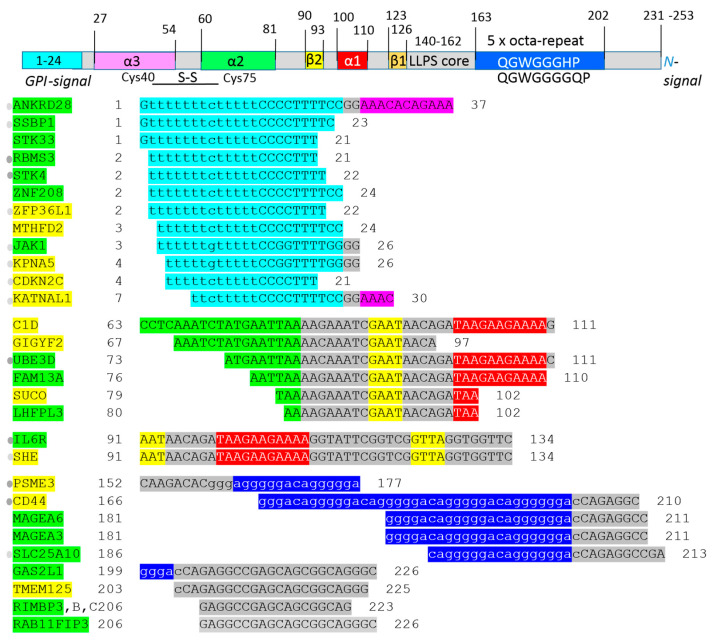
Identified genes/proteins via 1-L transcription of *C*-(AA)n-*N* sequence. The found sequences are colored according to the schematic domain architecture of prion protein. α = alpha-helical domains (magenta, light green, red), β = beta-sheet stretches (yellow), 5 x octa-repeat sequence (blue) and GPI-signal sequence (cyan). A GPI (glycosylphosphati-dylinositol) anchor is added at AA 230 as a post-translational modification. An LLPS core means that the main sequence participated in liquid–liquid phase separation. Green highlights show alignments with the gene transcript RNA sequence (post-transcriptionally repressed) and yellow highlights show alignments with the reverse complement RNA sequences (post-transcriptionally promoted). The identified genes/proteins can be directly (●) or indirectly (●) linked to prion diseases.

The amino acid sequence of PrP^C^ was 1-L transcribed into the four nucleic acid sequences (see Materials and Methods) and then the nucleic acid sequences were applied in BLASTn process screening. 1-L transcription and BLASTn alignments generated both the mRNA sequence (plus/plus strand) and the reverse complementary RNA sequence (plus/minus strand). Protein sequences that were 1-L compatible with a reverse complementary RNA sequence were considered as sequences evolved to recognize regulatory micro-RNAs (miRNAs). These small endogenous RNAs pair and bind to mRNA sites to induce post-transcriptional repression. Reducing the level of miRNAs or other small regulatory RNAs can thus promote translation. Alignment with reverse complement RNA sequences (plus/minus strand) is considered to be promotive (yellow in the figures). In contrast, alignment with the RNA sequence of the gene (plus/plus strand) is considered to be repressive (green in the figures), since the sequestering and blocking of free mRNA by the test protein represses translation. The genes identified according to these alignments are listed in Figure 3 (1-L transcription of N-(AA)n-C sequence) and Figure 4 (1-L transcription of C-(AA)n-N sequence). The main finding of this analysis is that 53.2% of the identified genes/proteins can currently be directly or indirectly associated with the prion diseases discussed in Section 2.1. Genes/proteins that map the relationship between prion protein and mitochondria are discussed in Section 2.2. Genes/proteins that are associated with cancer are discussed in Section 2.3. The genes/proteins that define the relatedness between PrP^C^ and COVID-19 are discussed in Section 2.4. Genes/proteins involved in ER stress and potentially regulated by PrP^C^ are discussed in Section 2.5.

As mentioned in the introductory section, the GGA triplet repeat RNA aptamer (R12) can protect against the misfolding of the normal prion protein (PrP^C^) into its abnormal form (PrP^Sc^). Years ago, it was shown in experiments with ScN2a cells that R12 binds to the N-terminal half of PrP^C^ and inhibits the formation of PrP^Sc^ [13]. This RNA aptamer was used as an example in the introductory section to familiarize the reader with the proposed method of 1-L transcription. The PrP^C^ octa-repeat sequence is 1-L compatible with R12 (Figure 1A). The R12 RNA aptamer is just one example found in the literature that validates the proposed method. The crystal structure of the ribosomal release factor 1 (RF1), which interacts with P-site tRNA (4V63), can be another example that explains the principle of the method (Figure 1B). The presented bioinformatics study using the still-new method of “1-L transcription” identifies genes/proteins associated with prion diseases and the spectrum of genes provides new insight into prion protein function. For example, the GPI signal peptide (GPI-SP) could be an important intracellular regulator in PrP^C^-producing cells, since the largest group of genes was identified in regions of GPI signal peptide alignment (Figure 3 and Figure 4). Currently, PrP^C^ is recognized mainly as a GPI (glycosylphosphatidyl-inositol)-anchored membrane protein whose α/β/γ/-cleavage or shedding (Figure 2B) leads to extracellular signaling [25,26].

### 2.1. 1-L Transcription of the Prion Protein and Genes/Proteins Identified as Being Relevant to Prion Diseases

1-L transcript and BLASTn alignments of the human prion protein revealed 47 genes/proteins, of which 53.2% may be directly or indirectly associated with prion diseases (Figure 3 and Figure 4). One example is post-transcriptionally promoted CD44 (Figure 4), which enhances ER stress resistance in a UPR-dependent manner, and also improves longevity [27]. Furthermore, CD44 is expressed in the regions of the brain showing astrocytic prion accumulation [28]. Post-transcriptionally promoted PSME3 (Figure 4), which is the 11S activator of the 20S proteasome catalytic core, may be associated with increased proteasome functionality and with an interaction between 20S proteasomes and prion proteins [29]. The post-transcriptional repression of the dicarboxylate carrier SLC25A10 (Figure 4), which cooperates with the circadian protein CLOCK and regulates rhythmic mitochondrial respiration [30], may be associated with sleep alterations and electroencephalographic changes in sCJD patients [31]. Interestingly, these three genes/proteins show 1-L compatibility with the octa-repeat (Figure 4).

The PrP^c^ α1-β2 region and part of the α2 region have 1-L promoting compatibility with IL1RAP (interleukin 1 receptor accessory protein, Figure 3). Moreover, the IL-1 signaling pathway was proposed as a possible therapeutic target for prion disease, since IL-1R-deficiency significantly delayed the onset of neurodegeneration in mice [32,33]. The PrP^c^ α1-β2 region also showed post-transcriptional promotion of TULP4 (Figure 3), which was listed amongst 52 prion strain-discriminating genes [34].

The PrP^c^ α1-β1 reverse direction showed 1-L repressive compatibility with IL6R (Figure 4, α1-β1). IL-6/IL6R signaling is involved in astrogliosis, which is a hallmark of prion diseases [35]. The same sequence also showed the promotion of the SH2 domain-containing adapter protein SHE (Figure 4), which is an evolutionarily conserved inhibitor of c-Abl kinase [36]. A neurotoxic prion fragment (PrP106-126) has been shown to activate IL6R [35] and c-Abl [37]. c-Abl triggers the upregulation of STK4/MST1 [37], and the GPI-signal sequence of the pro-prion protein showed post-transcriptional repression of STK4/MST1 (Figure 4). However, pro-death STK4/MST1 signaling is activated in prion disease models [37,38]. Hypothetically, when PrP106-126 initiates aggregation, then prion protein in LLPS-condensates is reduced and sequence-specific PrP–RNA interactions are not provided.

The GPI-signal peptide (GPI-SP) sequence showed a repression of mitochondrial SSBP1 (Figure 4), which protects cells from proteotoxic stresses by potentiating the stress-induced transcriptional activity of HSF1. HSF1 is known to confer an important protective function against prion diseases [39]. In contrast, the GPI-SP sequence promotes ZFP36L1/BRF1 (Figure 4), which is an ARE-RBP involved in 3′UTR regulation of mRNA decay [40]. 14-3-3 has a central chaperone-like function [41] and binds to phosphorylated ZFP36L1/BRF1 to inhibit its mRNA decay activity [42]. Increased 14-3-3 expression is a reliable marker of sCJD [43]. The promotion of the importin KPNA5 (Figure 4, GPI-SP) plays a role in IFN-signaling [44], thereby interfering with prion propagation. Moreover, some IFN-stimulated genes might have protective roles in the brain [45]. KPNA5 can also act as a chaperone by shielding aggregation-prone proteins from misfolding, resulting in irreversible phase-transition into insoluble aggregates [46]. Promoted CDKN2C (Figure 4, GPI-SP) binds to CDK4 or CDK6 to reduce CDK activation, thereby controlling the progression of the cell cycle through G1 [47]. Cyclin D1-CDK4/6 activity is generally reduced during the late G1 phase, whereas cyclin E-CDK2 activity increases and promotes the G1/S transition by phosphorylating RB1. Some cancers therefore upregulate CDKN2C expression to activate CDK2 and cell cycle progression [48]. Interestingly, the pathological course of prion disease is characterized by the progressive proliferation of microglial and astrocyte populations [49].

Promoted KATNAL1 (Figure 4, GPI-SP) is a catalytic subunit of the microtubule-severing enzyme katanin. TAU protects microtubules against katanin [50], and the concentration of TAU in the cerebrospinal fluid of CJD patients is markedly increased [51]. Interestingly, PrP^c^ and Aβ peptide may have opposite effects, since 1-L transcription of the Aβ peptide identified post-transcriptional repression of KATNAL1 [3]. Consequently, PrP^c^ misfolding may initiate Aβ accumulation and misfolding. Coexistent amyloid Aβ peptide plaques have been reported in some transmissible spongiform encephalopathies in what could be a widespread phenomenon [52].

Repressed ANKRD28 (Figure 4, GPI-SP) is a scaffolding subunit of the heterotrimeric protein phosphatase PP6 complex (ANKRD28-PP6R1,2-PP6c) [53]. In the Hippo pathway, the PP6 complex is likely to compete with MST1 for binding to MOB1 and for its phosphorylation/dephosphorylation [53]. Differential editing characterized by reverse-frequency alterations between sCJD and a mouse model of epilepsy was observed at the 3′UTR of Ankrd28 [54].

Repressed DAPK2 (Figure 3, α2) is a Ca^2+^/CaM-regulated serine/threonine kinase that can suppress mTOR activity to induce autophagy [55]. DAPK2 also phosphorylates BECLIN-1, a core protein in the autophagic machinery, leading to the dissociation of its inhibitor, BCL-XL [56]. Autophagy is known to be decreased in scrapie disease [57,58]. Repressed DYM (Figure 3, α3-GPI-SP) is involved in Golgi organization [59], and fragmented Golgi and TGN are observed in CJD [60]. Post-transcriptionally repressed OR6C4 (Figure 3, α3-GPI-SP), which belongs to a large olfactory receptor family, is transcriptionally increased in scrapie [61]. The repressed TF SOX4 (Figure 3, GPI-SP) is a downstream signaling target for the cytokine TGF-β and suppresses T_H_2 cell–mediated inflammation. During prion infection, SOX4 appears to be transcriptionally downregulated [62].

### 2.2. The Prion Protein and Mitochodria

As shown in Figure 3 and Figure 4, the GPI-attachment signal peptide of PrP^c^ (GPI-SP) post-transcriptionally regulates an important number of the identified genes/proteins. When expressed as a cytosolic peptide, GPI-SP was observed to localize to the mitochondria and to induce mitochondrial fragmentation and vacuolization. This was followed by the loss of mitochondrial membrane potential, ultimately resulting in apoptosis [63]. PrP^C^ has also been localized to the inner mitochondrial membrane where it appears not to be attached to the membrane via the GPI anchor, but rather inserted into the membrane so that the C-terminus is protected [64]. Recently, a point mutation in GPI-SP was shown to accelerate the development of prion disease [65].

Post-transcriptionally repressed methyltransferase HEMK1 (Figure 3, α3-GPI-SP), which is similar to a bacterial enzyme, is localized in the mitochondria and is therefore known as mitochondrial protein release factor methylation C (MPRMC). HEMK1 methylates the glutamine residue of the GGQ motif of mitochondrial release factors (RFs) [66]. RFs recognize the stop codon and consequently the peptidyl-tRNA bond of the tRNA located in the P-site of the ribosome. The peptidyl-tRNA bond is cleaved to release the newly synthesized protein, thereby terminating mRNA translation. The glutamine (CAA codon) in the universally conserved GGQ motif (Figure 1B) is inserted into the center of the peptidyl transferase. In addition, it recognizes and interacts with the C_2452_A_2451_ of rRNA and the A_76_ of tRNA, and is positioned to contribute directly to peptidyl-tRNA hydrolysis and translation termination [16]. In bacteria, the glutamine methylation of the GGQ motif of RFs by the enzyme PrmC is essential for translational termination and transcript recycling [66]. The GGQ motif and PrP^c^ octa-repeat are 1-L transcribed to GGA RNA (Figure 1), such that the GGA triplet repeat RNA binds PrP^C^ and reduces the PrP^Sc^ level [13].

Post-transcriptionally repressed SSBP1 (Figure 4, GPI-SP) is essential for the initiation of mitochondrial DNA replication [67,68]. Post-transcriptionally promoted MTHFD2 (Figure 4, GPI-SP) participates in the mitochondrial part of folate-mediated one-carbon metabolism [69]. Impaired one-carbon metabolism is involved in neurodegeneration [69]. Repressed Hippo kinase STK4/MST1 (Figure 4, GPI-SP) is activated in macrophages during infection. This signaling not only contributes to the anti-microbial process by promoting ROS generation, but also protects macrophages against oxidative stress by limiting the production of ROS [70]. The release of ROS leads to the recruitment of STK4/MST1 from the cytosol to the mitochondrial membrane [70].

Promoted GSTO2 (Figure 3, GPI-SP) exhibits thioltransferase activity, which may have a role in regulating GSH levels [71]. PrP^C^ shows a protective effect during oxidative stress, with the GSH level in PRNP^−/−^ thymocytes being significantly decreased [72]. However, the overexpression of GSTO2 suppresses mitochondrial respiration [71]. Post-transcriptionally repressed dicarboxylate carrier SLC25A10/DIC (Figure 4, octa-repeat), together with SLC25A11 (oxoglutarate carrier), are the main cytoplasmic GSH transporters across the mitochondrial inner membrane [73]. SLC25A10/DIC supports both redox- and energy-homeostasis, significantly improves cell injury and mitochondrial dysfunction and inhibits the mitochondrial apoptosis pathway [74]. Mitochondrial respiration and ROS production show rhythmic activity, while the circadian protein CLOCK regulates cell metabolism via the mitochondrial carrier SLC25A10 [30]. Sleep alterations and electroencephalographic (EEG) changes characterize sCJD [31].

### 2.3. The Prion Protein and Cancer

A link between PrP^C^ and cancer progression was first reported more than 20 years ago in pancreatic cancer cells [75]. PrP^C^ is now known to be associated with diverse solid cancer types, such as gastric cancer and glioma [76]. The ectopic expression of PrP^C^ in gastric cancer cells was shown to promote cell proliferation and the G1/S transition [77]. Transfection with PrP^C^ increased the transcription of CyclinD1 (CCND1) and CDK4, while CCND1 also increased at the protein level [77]. The present bioinformatics study showed that the 1-L transcription of PrP^C^ (PRPN) leads to the post-transcriptional promotion of CDKN2C and ZFP36L1 (Figure 4, GPI-SP). The cyclin-dependent kinase inhibitor 2C (CDKN2C, INK4C, p18) binds to CDK4/6 and reduces CDK-kinase binding/activation to CCND1, which controls G1-S progression [47]. ZFP36L1 is an ARE-RBP that binds to the 3′UTR to mediate mRNA decay, including the mRNAs for CCND1 [40] and CDK6 [78]. In light of this, both CDKN2C and ZFP36L1 are thought to protect against aberrant cell cycle progression. Nevertheless, the cell cycle is a tightly regulated process, and abnormal progression can be achieved through the upregulation of cell cycle inhibitors. Some cancers upregulate CDKN2C [48], and it has been suggested that ZFP36L1 promotes gastric cancer [79]. Moreover, when PrP^C^ post-transcriptionally inhibits CCND1-CDK4/6, the cell may nevertheless transcriptionally overexpress CCND1-CDK4/6. This may explain why PrP^C^ transfection abnormally increases the transcription of CCND1 and CDK4 genes, leading to abnormal cell cycle progression [77].

SSBP1 suppresses TGF-β-driven EMT by regulating mitochondrial retrograde signaling [80]. However, PrP^c^ post-transcriptionally represses SSBP1 (Figure 4, GPI-SP). The subsequent loss of SSBP1 decreases mitochondrial DNA copy number, thereby activating TGF-β promoter activity [80]. On the other hand, repressed TF SOX4 (Figure 3, GPI-SP) is a downstream signaling target for cytokine TGF-β and is required for TGF-β-induced EMT [81].

Repressed RNA-binding protein RBMS3 (Figure 4, GPI-SP) plays a significant role in many diseases, especially cancer initiation and progression. The absence of RBMS3 activates the Wnt/catenin pathway, with low RBMS3 expression usually correlating with worse prognosis [82], as, for example, in gastric cancer [83]. PrP^c^ post-transcriptionally represses FAM13A (Figure 4, α2-α1), with the reduced level of FAM13A protein leading to accelerated epithelial cell proliferation in murine lungs during the recovery phase after smoking/infection-induced injury [84].

PrP^C^ post-transcriptionally promotes CD44 (Figure 4, octa-repeat). The single CD44 gene codes for a large family of multifunctional, single-chain, transmembrane glycoproteins belonging to the class of cell adhesion molecules (CAMs). CD44 has been recognized as a cancer stem cell marker in several tumor types. The binding of CD44 to its ligands osteopontin (OPN) and hyaluronan (HA) induces stemness and EMT in glioblastomas and breast cancer. In colorectal cancer, OPN and CXCL12 induce CD44v6 expression. The subsequent collaboration between CD44v6, HGF and Met induces EMT [85].

PrP^C^ post-transcriptionally promotes the hedgehog acyltransferase HHAT (Figure 3, α2). This catalyzes the transfer of a palmitoyl lipid to the N-terminal cysteine of Hedgehog (HH) precursor proteins. HH precursor proteins must undergo N-terminal palmitoylation before they can function as HH signaling ligands (Sonic, Desert, and Indian) [86]. The excessive expression of HH signaling molecules (ligand-dependent signaling) may lead to cancer, and several inhibitors of HH signaling pathways have been developed for cancer treatment [87].

PrP^C^ post-transcriptionally represses STK4/MST1 (Figure 4, GPI-SP), resulting in the nuclear localization of YAP/TAZ (Hippo signaling pathway). This interferes with the cell cycle and increases the expression of pro-cancerous genes [88].

### 2.4. The Prion Protein and COVID-19

Almost 600 million people have been infected with SARS-CoV-2, with approximately half exhibiting some degree of continuing health complications. This is generically referred to as persistent post-COVID syndrome, or long COVID. Up to 35% of all elderly COVID-19 patients also develop mild-to-severe encephalopathy due to complications arising from a SARS-CoV-2-induced cytokine storm [89]. COVID-19 is strongly associated with the symptomology, onset, and development of human prion diseases [89,90,91,92,93].

PrP^C^ post-transcriptionally represses IL6R (Figure 4, α1-β1), which is mostly a membrane-bound receptor. IL-6 activates cells by binding to membrane-bound IL6R and subsequently forming a glycoprotein 130 (gp130) homodimer. Cells that express gp130, but not IL6R, can be activated by IL-6 and soluble IL6R (sIL6R) that is shed from the cell surface. Signaling by the IL-6/sIL6R complex is referred to as “trans-signaling” and promotes the inflammatory response by inducing leukocyte activation and survival, as well as by affecting tissue permeability [94]. Soluble gp130 (sgp130) is present in the blood, and the balance of IL-6, IL-6/sIL6R and IL-6/sIL6R/sgp130 complexes allows simultaneous classic- and trans-signaling [95]. Interestingly, the depletion of cellular cholesterol triggers the shedding of human IL6R by ADAM10 [96]. Previous “1-L transcription” studies showed that SARS-CoV-2 infection reduces cellular cholesterol [4,5]. Moreover, increased IL-6 is a marker of COVID19 [97,98], and may trigger IL-6/sIL6R trans-signaling. Interestingly, this signaling activates vascular inflammatory responses via HIF1α-induced glycolysis. The short-term inhibition of IL6R signaling in COVID-19 patients protects the vasculature from injury, while the persistent inhibition of IL-6 signaling increases the susceptibility to severe complications (bacterial/fungal secondary infections) and is associated with poor outcomes [99]. The protease ADAM10 is responsible for PrP^c^ shedding (Figure 2B) [25], and the shed PrP^C^ post-transcriptionally represses IL6R. Γ-cleavage of PrP^C^ and the consequent N3-fragment signaling (Figure 2B) will also repress IL6R (Figure 4, α1-β1). In addition, PrP^C^ promotes the ZFP36L1 RBP (Figure 4, GPI-SP) that mediates the mRNA decay of HIF1α [40]. In light of this, it appears that PrP^C^ may be upregulated upon SARS-CoV-2 infection to limit excessive signaling by IL-6. The upregulation of PrP^C^ in the presence of PrP^Sc^ may cause PrP^fibril^ and exacerbate prion diseases.

PrP^C^ is involved in finely regulating the redox balance. When exposed to oxidative stress, the GSH level in PRNP^−/−^ thymocytes decreases significantly compared to WT thymocytes [72]. The promoted GSTO2 (Figure 3, GPI-SP) exhibits thioltransferase activity, which may have a role in regulating GSH levels [71]. Polymorphisms in GSTO1 and GSTO2 have been associated with laboratory parameters of inflammation in COVID-19 [100,101].

PrP^C^ post-transcriptionally promotes KPNA5 (Figure 4, GPI-SP), which belongs to the importin-α family (IMPα6). TDP-43 and NF-κB are examples of cargo for KPNA5 [46]. KPNA5 also binds and transports STAT1 to the nucleus, which is a key step during IFN-signaling [44]. COVID-19 patients show alterations in KPNA5 [102]. Moreover, ivermectin is a potent inhibitor of SARS-CoV-2 and targets the IMPα component of the IMP α/β1 heterodimer [103].

PrP^C^ post-transcriptionally promotes PSME3 (Figure 4, octa-repeat), which is an activator of the 20S proteasome catalytic core. During viral infections, TLR ligands upregulate the expression of the 11S proteasome subunit PSME3 in macrophages via NF-κB-mediated transcription. PSME3, in turn, enhances the transcriptional activity of NF-κB by directly binding to and destabilizing KLF2, which is a negative regulator of NF-κB transcriptional activity [104]. Interestingly, the upregulation of the proteasome components (including PSME3) was observed in COVID-19 patients with hyperinflammatory conditions [105].

PrP^C^ post-transcriptionally promotes GIGYF2 (Figure 4, α2-β2), which interacts with 4EHP (5′ cap-binding protein) to form the GIGYF2–4EHP translational repressor complex. The SARS-CoV-2 protein NSP2 impairs the silencing capacity of the human 4EHP–GIGYF2 complex [106].

### 2.5. The Prion Protein and ER Stress

The accumulation of unfolded proteins leads to ER stress. This is followed by an adaptive response via the activation of several pathways including the UPR, PKR-like ER kinase (PERK), inositol-requiring transmembrane kinase/endoribonuclease 1 (IRE1) and activating transcription factor 6 (ATF6). The association between ER stress, UPR and neuropathology is well established, and prolonged ER stress activates apoptosis signaling, leading to neuronal death. Prion diseases were shown to involve mainly the PERK pathway, as well as the IRE1 and ATF6 pathways [107]. Persistent ER stress appears to cause a deficiency of the GPI anchor (ATF6 pathway), which is required for the conversion of pro-prion proteins to GPI-anchored prion proteins [108].

PrP^C^ post-transcriptionally represses JAK1 (Figure 4, GPI-SP), thereby contributing to ER-stress-induced inflammation. JAK1 interacts with and phosphorylates PERK and mediates the ER stress-induced activation of STAT3, leading to IL-6 production [109]. PrP^C^ post-transcriptionally represses SSBP1 (Figure 4, GPI-SP). SSBP1 is a mitochondrial single-stranded DNA-binding protein that protects cells from proteotoxic stresses by increasing the transcriptional activity of stress-induced HSF1. HSF1 recruits SSBP1 to the promoters of genes encoding cytoplasmic, nuclear and mitochondrial chaperones, thereby increasing their transcription [110]. PrP^C^ post-transcriptionally represses RIC8B (Figure 3, α3). This chaperone regulates the folding and cellular abundance of heterotrimeric G protein α subunits, especially Gα_s/olf_. Olfactory neurons that lack RIC8B, and consequently Gα_olf_, are more susceptible to cell death [111].

PrP^C^ post-transcriptionally promotes KPNA5 (Figure 4, GPI-SP), the karyopherin subunit alpha 5. In addition to its classical functions in the nuclear import and export of cargo (e.g., TDP-43 and NF-κB), KPNA5 can also act as a chaperone by shielding aggregation-prone proteins against misfolding, accumulation and irreversible phase-transition into insoluble aggregates [46]. PrP^C^ post-transcriptionally promotes SUCO (Figure 4, α2-α1), an ER protein that participates in non-glycoprotein quality control. It does this via the SUCO/SLP1-TAPT1 complex, which binds unfolded proteins and protects them from degradation during folding. In the absence of SUCO, approximately 20–30% of newly synthesized proteins that would otherwise fold are degraded [112]. PrP^C^ post-transcriptionally promotes CD44 (Figure 4, octa-repeat), which increases basal ATF6 activity and ER-stress resistance [27].

## 3. Materials and Methods

The primary structures of proteins are involved in protein-RNA recognition/interaction. These processes are driven by 1-L and 2-L codes conserved in the amino acid codons (Figure 1). Such codes can be used to identify mRNA and miRNA sequences compatible with genes/proteins that are post-transcriptionally regulated by specific RBPs.

### 3.1. 1-L Transcription Procedure

**Figure 5:**
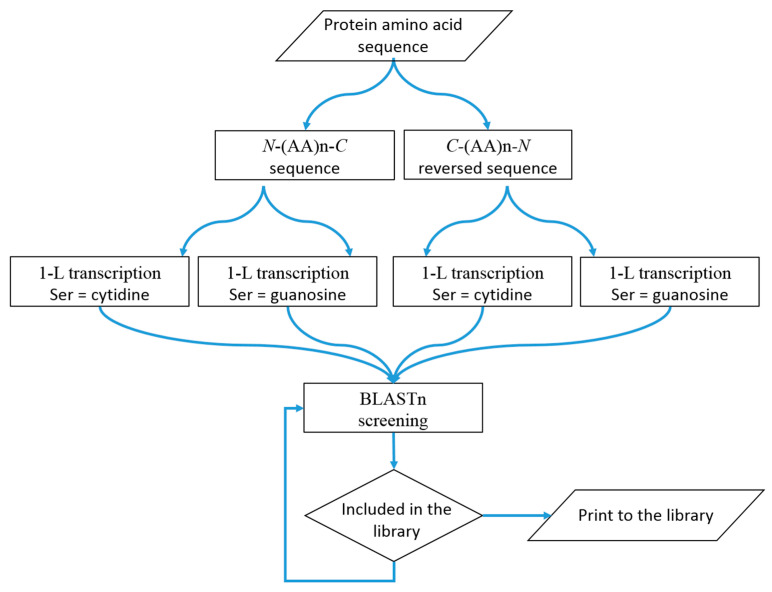
1-L transcription method. A schematic diagram that visually represents the steps and concept.

The 1-L transcription procedure is relatively simple and involves the amino acid sequence of the specific RBP being transcribed into an RNA sequence based on the nucleotide at the second position of the amino acid codon (1-letter code). The resulting nucleotide sequence is then used for the classical BLASTn screening of the human transcriptome. Reading of the 5’-RNA by the RBP can be carried out in both directions using the amino acid sequence *N*-(AA)n-*C* or the reverse amino acid sequence *C*-(AA)n-*N* (Figure 1). Hence, the 1-L transcription should be written for two amino acid sequences: one for *N*-(AA)n-*C* and the other for *C*-(AA)n-*N*. Serine (Ser, S) has two types of codons, one with C (cytidine) at the second position in the amino acid codon and the other with G (guanosine). Thus, two nucleotide sequences are obtained for each amino acid sequence, one with S-C-transcription and the other with S-G-transcription. A total of four nucleotide sequences are obtained (Figure 5).

### 3.2. BLASTn Screening Process

The BLASTn screening of the human transcriptome was performed as a standard nucleotide blast at NCBI (https://blast.ncbi.nlm.nih.gov/Blast.cgi) (accessed on 9 May 2024). This was performed separately for the four nucleotide sequences. The following parameters were used for this search: “Genomic + transcript databases” and “human genomic plus transcript”, “somewhat similar sequences” (blastn), word size = 7, maximum number of target sequences = 500 and expected threshold = 100.

## 4. Conclusions

Prion diseases are caused by the misfolding of a normal α-helix-rich PrP^C^ into an abnormal β-sheets-rich PrP^Sc^ (Figure 2A). PrP^C^ is an RBP (Figure 1A) and in theory may play a neuroprotective role inside biomolecular condensates. The sequence-specific RBP–RNA interactions within neurons are maintained inside LLPS-condensates [9] and preserve the protein-RNA recognition code (Figure 1B) [6,7]. RBPs use at least one amino acid sequence that can be 1-L transcribed into the exact nucleotide sequence of the recognized RNA. This can be used to identify genes/proteins that are post-transcriptionally regulated by the RBP. For example, based on this theory, PrP^C^ recognizes GGA triplet repeats (R12) using its octa-repeat sequence, and interacts with R12 using Lys-rich sequences [13] located on either side of octa-repeat sequence (Figure 1A).

The 1-L transcription of the human prion protein and BLASTn alignments resulted in the identification of a set of genes/proteins (Figure 3 and Figure 4), of which approximately half were directly or indirectly associated with prion diseases (discussed in Section 2.1). Many of the identified genes/proteins were found to be involved/associated with mitochondria (discussed in Section 2.2), cancer (discussed in Section 2.3), COVID-19 (discussed in Section 2.4) and ER stress (discussed in Section 2.5). Furthermore, the GPI-attachment signal peptide (GPI-SP) post-transcriptionally regulates a considerable number of the identified genes/proteins (Figure 3 and Figure 4). For example, promoted KATNAL1 (Figure 4, GPI-SP) is a catalytic subunit of the microtubule-severing enzyme katanin. TAU protects microtubules against katanin [50], and the concentration of TAU in the cerebrospinal fluid of CJD patients is markedly increased [51]. Interestingly, PrP^c^ and Aβ peptide may have opposite effects, since 1-L transcription of the Aβ peptide identified the post-transcriptional repression of KATNAL1 [3]. Consequently, PrP^c^ misfolding may initiate Aβ accumulation and misfolding. Coexistent amyloid Aβ peptide plaques have been reported in some transmissible spongiform encephalopathies in what could be a widespread phenomenon [52].

Interestingly, this method identified the mitochondrial methyltransferase HEMK1 (Figure 3), which methylates the glutamine residue of the GGQ motif of mitochondrial release factors, which is essential for translation termination and transcript recycling [66]. GGQ motif and PrP^c^ octa-repeat are 1-L transcribed to GGA RNA (Figure 1). GGA triplet-repeat RNA was found to reduce the PrP^Sc^ level in mouse neuronal cells persistently infected with a transmissible spongiform encephalopathy agent [13]. The same GGQ amino acid motif was proposed for the recognition of ribose in prebiotic history, with ribose being the sugar component of RNA [17].

## Data Availability

Data are contained within the article.
